# Conjunctival Medial Tarsorrhaphy for Lower Eyelid Medial Ectropion: A Simple and Effective Technique

**DOI:** 10.7759/cureus.96620

**Published:** 2025-11-11

**Authors:** Saori Kotaki, Tomoyuki Kashima

**Affiliations:** 1 Ophthalmology, Oculofacial Clinic Group Tokyo, Tokyo, JPN

**Keywords:** conjunctival medial tarsorrhaphy, epiphora, lacrimal drainage system, lower eyelid medial ectropion, oculoplastic surgery, punctal malposition

## Abstract

Introduction

We developed a novel technique, conjunctival medial tarsorrhaphy (CMT), to treat medial ectropion through a minimally invasive, skin-sparing approach. We evaluated the efficacy and safety of CMT in patients with lower eyelid medial ectropion (LEME).

Methods

We retrospectively analyzed 22 eyelids from 18 patients who underwent CMT between June 2018 and August 2024. The procedure involved conjunctival resection nasal to the puncta and direct skin approximation without skin incisions. Clinical outcomes included changes in margin reflex distance 2 (MRD2), vertical distance between the puncta, and superficial punctate keratopathy (SPK) score. Adverse events were also recorded.

Results

The mean age was 71±14 years. LEME etiologies included involutional (five cases), facial nerve palsy (five cases), previous surgery (seven cases), and trauma (one case). Concomitant procedures were performed in some cases. The mean follow-up was 6.5±4.6 months. MRD2 decreased (improved) from 8.99±1.77 mm to 7.07±1.57 mm (p<0.001), punctal distance from 4.21±1.12 mm to 1.70±1.10 mm (p<0.001), and SPK score from 3±2 to 1±2 (p<0.001). Minor complications included two canalicular epithelial injuries, managed successfully with silicone intubation.

Conclusions

CMT is a simple, minimally invasive, and reproducible technique that provides effective LEME correction with improvements in eyelid position and ocular surface health. It can be safely repeated and may serve as a first-line surgical option for LEME. Further studies are warranted to evaluate long-term outcomes and effects on lacrimal function.

## Introduction

Lower eyelid medial ectropion (LEME) is a relatively uncommon but functionally and aesthetically significant eyelid malposition. It is characterized by eversion of the medial aspect of the lower eyelid away from the globe, primarily resulting from laxity or degeneration of medial canthal support structures and dysfunction of the orbicularis oculi muscle and lower eyelid retractors. The causes of LEME include involutional changes, facial nerve palsy, thyroid eye disease, postoperative scarring, trauma, and chronic dermatitis [1,2]. LEME can result in punctal eversion and incomplete eyelid closure, leading to ocular surface dryness, superficial punctate keratopathy (SPK), epiphora, photophobia, foreign body sensation, and cosmetic concerns [3,4].

Various surgical techniques have been proposed to correct LEME, including medial spindle conjunctivoplasty [5], the lazy-T and modified lazy-T procedures [6-8], medial lid tightening [9], medial tarsal suspension [10], and medial canthal tendon plication [11,12]. However, many of these procedures are limited by insufficient vertical lifting force or require technically demanding dissection near the canaliculi [10]. The classic lazy-T method may lead to improper punctal-globe apposition [3]. Medial spindle techniques can offer mild punctal repositioning, but they may be insufficient for correcting medial canthal laxity [5].

To address these limitations, we developed a novel technique, namely conjunctival medial tarsorrhaphy (CMT). CMT involves resection of the palpebral and bulbar conjunctiva nasal to the puncta, followed by direct approximation of the upper and lower eyelid margins without skin incision. This mucosal-side adhesion promotes vertical tightening and functional and structural correction in a relatively simple manner while preserving the skin and canalicular anatomy. In this retrospective study, we evaluated the efficacy and safety of CMT for the treatment of LEME.

## Materials and methods

**Figure 1:**
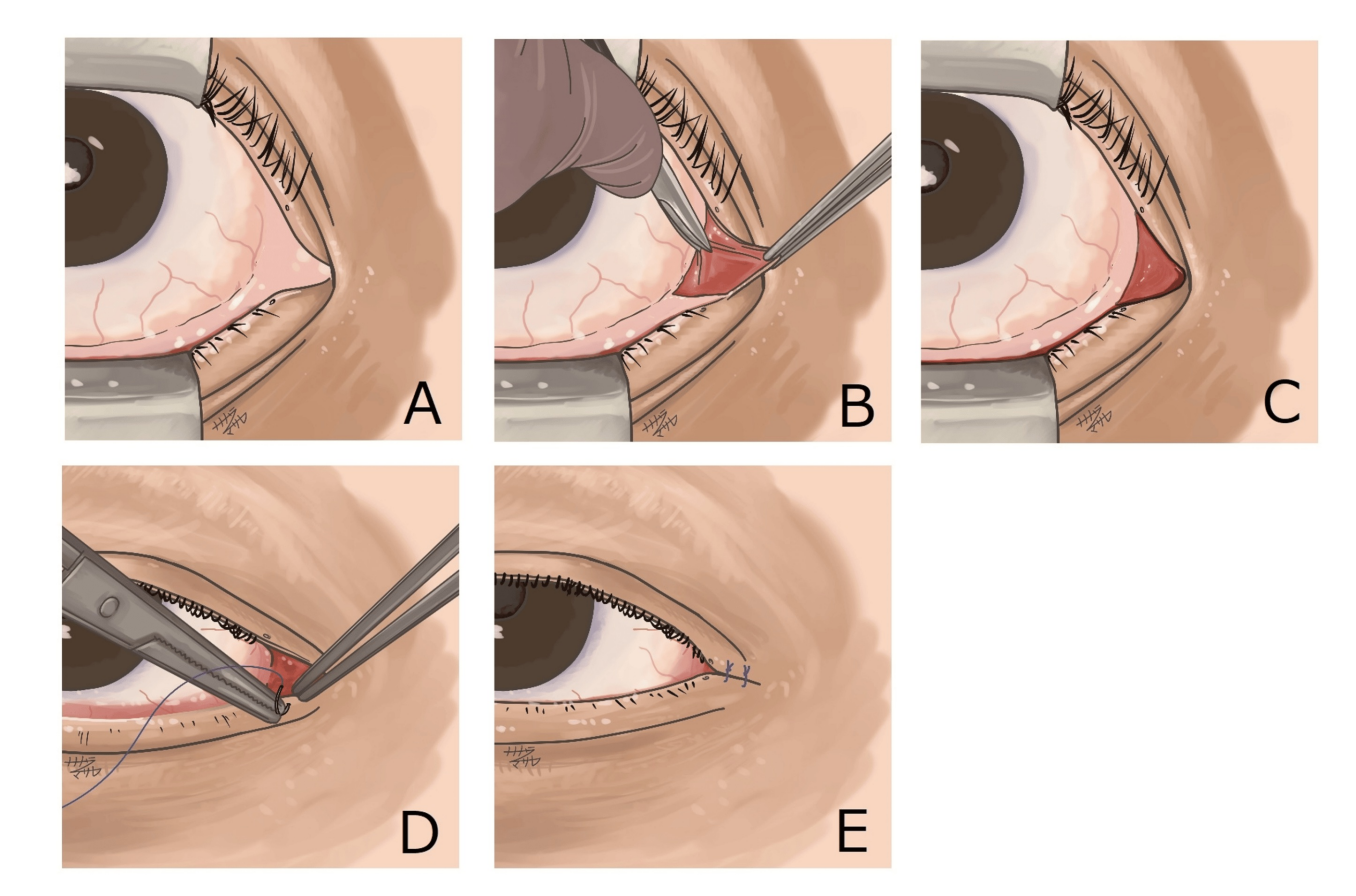
Intraoperative steps of the CMT technique. (A, B) Resection of the palpebral and bulbar conjunctiva nasal to the puncta, including the caruncle. (C) Completion of conjunctival resection, exposing the underlying tissue. (D) Approximation and suturing of the upper and lower eyelid skin margins nasal to the puncta using Vicryl. (E) Final appearance at the end of surgery, showing mucosal adhesion. All photographs are original and owned by Oculo Facial Clinic Tokyo (surgical photo archive: 2018-2024). CMT: conjunctival medial tarsorrhaphy.

CMT was performed under local anesthesia as follows: (1) resection of the palpebral and bulbar conjunctiva nasal to the upper and lower puncta, including the caruncle; and (2) direct approximation of the skin margins nasal to the puncta with two interrupted 6-0 polyglactin 910 (Vicryl) sutures (absorbable; no suture removal required). Representative images of the surgical steps are shown in Figures 1A-1E.

We retrospectively reviewed 22 eyelids from 18 patients who underwent CMT for LEME between June 2018 and August 2024 at our institution. The unit of analysis was the eyelid. The following parameters were evaluated: age, sex, LEME etiology, presence of concurrent procedures, and adverse events. Inclusion criteria were clinically diagnosed LEME and availability of standardized pre- and postoperative photographs; eyelids with inadequate imaging or missing outcome data were excluded. Concomitant procedures performed in the same session (e.g., lateral canthopexy, lateral sling, skin graft) were recorded a priori and are reported descriptively. Surgical outcomes included changes in margin reflex distance 2 (MRD2), vertical distance between the upper and lower puncta, and severity of superficial punctate keratopathy (SPK). Postoperative outcomes were assessed at the latest available follow-up.

Photographs were taken pre- and postoperatively under standardized conditions using the same magnification, lighting, and distance, and analyzed using ImageJ software (Bethesda, MD: National Institutes of Health; public domain) to calculate MRD2 and punctal distance based on the ratio to corneal diameter, assuming an average horizontal corneal diameter for the Japanese population (12.17 mm for males, 11.95 mm for females) [13]. SPK was graded using the area-density (AD) classification based on fluorescein staining [14]. The AD classification is a published clinical method and does not require a commercial license. For analysis, we used a composite AD score defined a priori as A (area: 0-3) + D (density: 0-3), with a total of 0-6; higher values indicate more severe SPK. Preoperative and postoperative measurements were compared using the paired t-test. Two-tailed tests with α=0.05 were used. Statistical analyses were performed using Microsoft Excel 2016 (MSO build 2508; Redmond, WA: Microsoft Corp.). No proprietary or licensed software or scoring systems were used in this study.

This study was approved by the Ethics Committee of Oculofacial Clinic Tokyo, on June 27, 2025 (IRB approval number: 2025062701). The study adhered to the tenets of the Declaration of Helsinki, as amended in 2013. Written informed consent was obtained from all patients for the use of their clinical data and identifiable photographs. The signed consent forms are on file and have been archived by the authors.

## Results

The mean age of the patients was 71±14 years, with a male:female ratio of 5:4. The etiology of LEME was involutional in five cases, facial nerve palsy in five, previous surgery in seven, and trauma in one case. Concomitant procedures included canthopexy (four eyelids), lateral tarsal strip (11 eyelids), and skin grafting (three eyelids). The mean postoperative follow-up period was 6.5±4.6 months.

Adverse events included one case of conjunctival edema and two cases of intraoperative canalicular epithelial injury. Conjunctival edema resolved spontaneously within three months. In patients with canalicular injury, silicone tube intubation was performed intraoperatively, and the postoperative course was uneventful (Table 1).

**Table 1:** Summary of patient characteristics and surgical details. LEME: lower eyelid medial ectropion; LTS: lateral tarsal strip procedure.

Parameters	Values
Age (years)	71±14
Sex	Male:female = 5:4
Follow-up period (months)	6.5±4.6
Revision surgery	Six eyelids (27%)
Etiology of LEME	Involutional: 5, facial nerve palsy: 5, post-blepharoplasty: 7, trauma: 1
Combined procedures	Canthopexy: 4 eyes, LTS: 11 eyes (+ skin graft: 3 eyes)
Adverse events	Canalicular epithelial injury: 2 eyes (treated with silicone intubation, good outcome), conjunctival edema: 1 eye

**Figure 2:**
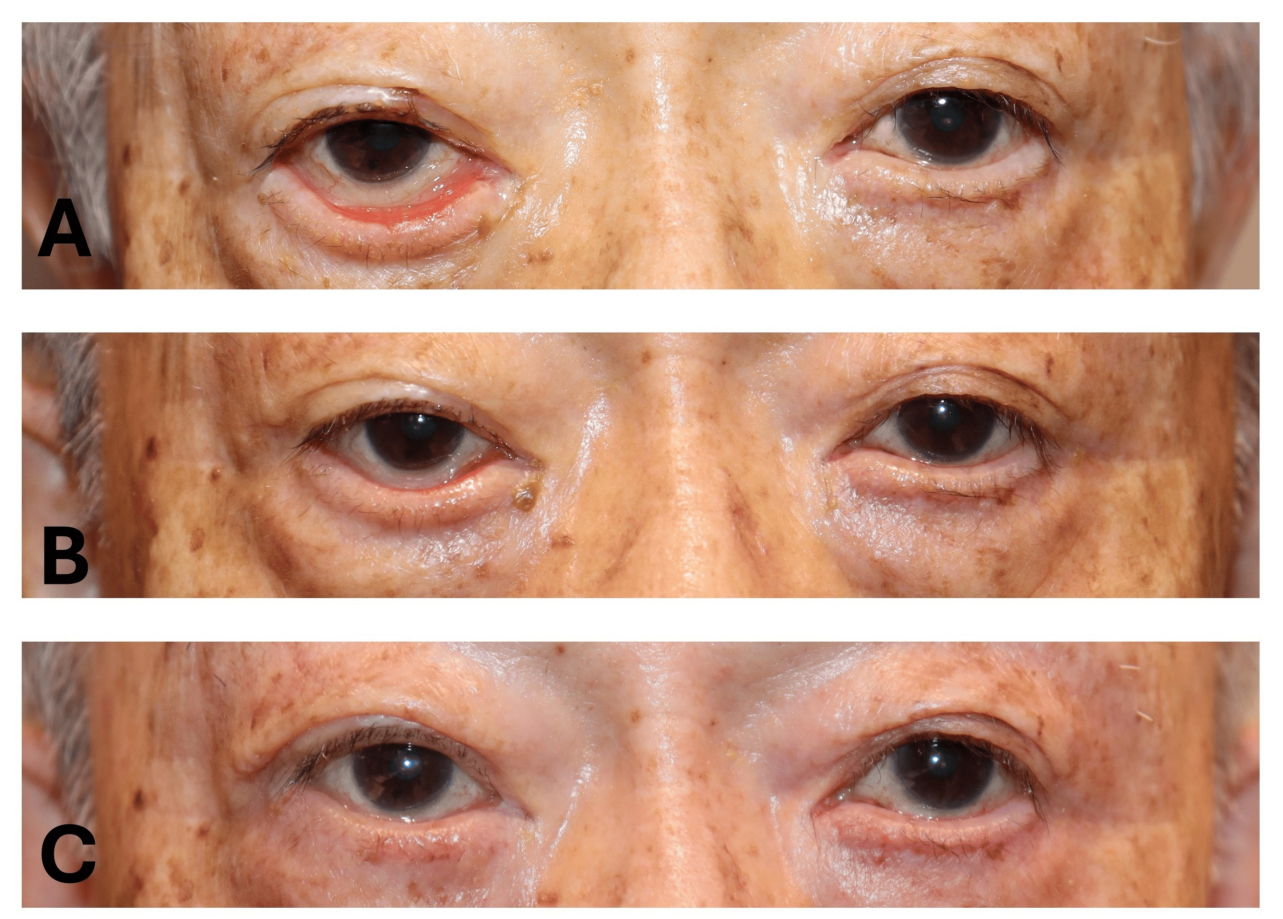
Clinical photographs before and after surgery of Case 1. A 91-year-old man with involutional right LEME. (A) The preoperative photograph shows right medial ectropion and displacement of the lower puncta. (B) Three months after the first surgery (CMT combined with canthopexy), partial improvement was observed, although mild residual LEME remained. (C) Three months after repeat CMT, complete correction of the medial eyelid malposition was achieved, with well-aligned puncta and improved lid-globe apposition. CMT: conjunctival medial tarsorrhaphy; LEME: lower eyelid medial ectropion

**Figure 3:**
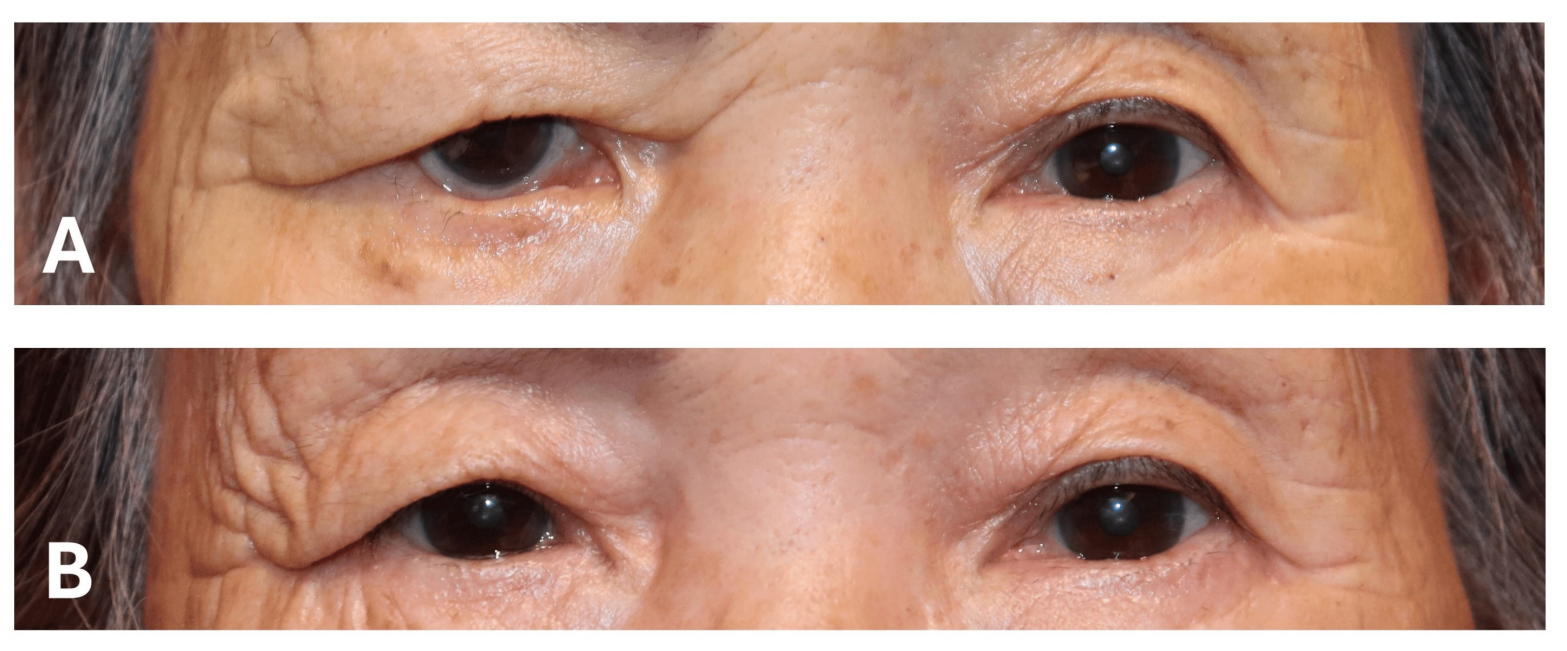
Clinical photographs before and after surgery of Case 2. (A) An 81-year-old woman with right facial nerve palsy. CMT and lateral tarsal strip were performed. (B) The postoperative image demonstrates substantial improvement in medial lid position. MRD2 decreased by 5.4 mm, punctal distance by 3.2 mm, and SPK score by five points (graded by the area-density classification [14]). CMT: conjunctival medial tarsorrhaphy; MRD2: margin reflex distance 2; SPK: superficial punctate keratopathy

**Figure 4:**
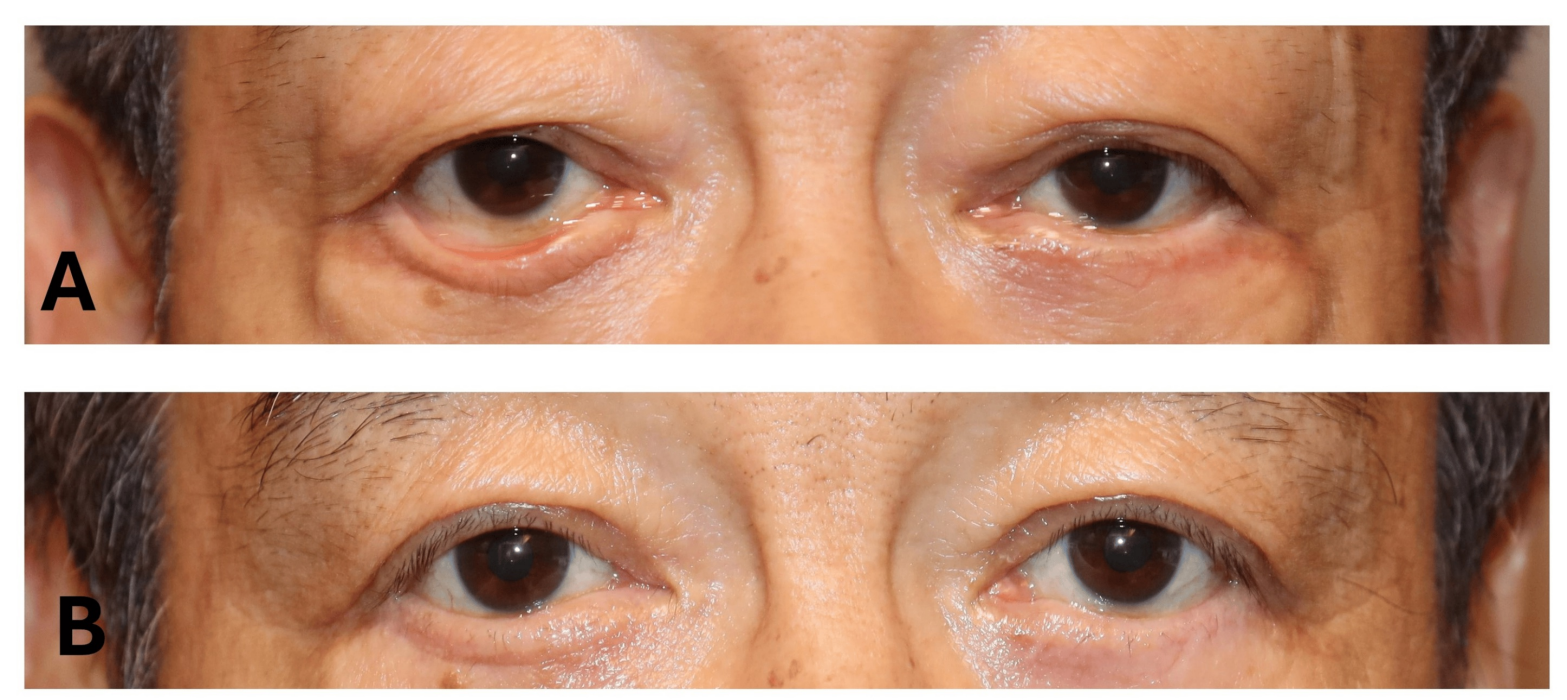
Clinical photographs before and after surgery of Case 3. (A) A 72-year-old man with postoperative LEME following previous lower blepharoplasty. CMT, LTS, and skin grafting were performed. (B) The postoperative appearance shows restored lid-globe apposition. MRD2 decreased by 4.2 mm, and the punctal distance decreased by 2.6 mm. SPK remained unchanged at 0 (graded by the area-density classification [14]). CMT: conjunctival medial tarsorrhaphy; MRD2: margin reflex distance 2; SPK: superficial punctate keratopathy; LTS: lateral tarsal strip procedure

MRD2 significantly decreased from 9.12±1.81 mm preoperatively to 6.69±1.25 mm postoperatively (mean change: 2.43±1.81 mm, p<0.001). Vertical punctal distance decreased from 4.15±1.17 mm to 1.45±0.93 mm (mean change: 2.69±1.28 mm, p<0.001), and SPK score decreased from 3±2 to 1±2 (mean change: 2±2, p<0.001), all indicating improvement (Table 2). Notably, additional conjunctival resection was performed on six eyelids. Representative cases are shown in Figures 2A-2C, 3A, 3B, 4A, 4B.

**Table 2:** Changes in MRD2, vertical punctal distance, and SPK score postoperatively compared with preoperatively. All parameters showed statistically significant improvements following CMT (p<0.001). Values are presented as mean±SD. Paired two-tailed t-tests were performed for 22 eyelids, showing significant improvements in MRD2 (t{21}=6.12, p<0.001), vertical punctal distance (t{21}=8.64, p<0.001), and SPK score (t{21}=4.69, p<0.001). CMT: conjunctival medial tarsorrhaphy; MRD2: margin reflex distance 2; SPK: superficial punctate keratopathy

Parameters	Preoperative	Postoperative	Mean difference	p-Value	T-value
MRD2	8.99±1.77 mm	7.07±1.57 mm	2.05±1.57 mm	<0.001	6.12
Vertical punctal distance	4.21±1.12 mm	1.70±1.10 mm	2.45±1.33 mm	<0.001	8.64
SPK score (area-density classification [14])	3±2	1±2	2±2	<0.001	4.69

## Discussion

This study demonstrates the anatomical, functional, and aesthetic efficacy of CMT for the treatment of LEME. LEME is a type of lower eyelid ectropion, but it is often refractory to lower eyelid retractor advancement or traditional lateral eyelid tightening procedures, such as canthopexy or lateral tarsal strip. In such cases, direct correction of the medial malposition is necessary. Lateral eyelid tightening, which corrects horizontal laxity, carries the risk of lateral displacement of the puncta and canalicular stretch when used alone [3]. Therefore, CMT may be a useful alternative.

LEME and punctal eversion were historically attributed to laxity of the medial canthal tendon, which was traditionally described as consisting of distinct anterior and posterior limbs anchoring the medial canthus to the anterior and posterior lacrimal crests, respectively [15]. However, a recent anatomical study suggested that the posterior limb of the medial canthal tendon may not exist as a discrete tendinous structure [16]. Instead, what was previously identified as the posterior limb is now understood to be a thick fibrous diaphragm comprising the fascia of Horner’s muscle and the lacrimal sac, which is structurally and functionally distinct from the true tendon. Accordingly, the primary medial stabilizers are now recognized to be Horner’s muscle and the medial rectus capsulopalpebral fascia.

Horner’s muscle, which arises from the posterior lacrimal crest and surrounds the lacrimal sac, plays a central role in maintaining punctal-globe apposition and lacrimal pump function. Degeneration or disinsertion of Horner’s muscle leads to punctal eversion and tear drainage disruption. Similarly, loosening or disinsertion of the medial rectus capsulopalpebral fascia, which acts as a dynamic retractor of the medial lower eyelid, allows the eyelid to sag and rotate outward, contributing directly to medial ectropion. Thus, LEME is now better understood to result from functional deterioration of Horner’s muscle and the medial rectus capsulopalpebral fascia, rather than medial canthal tendon laxity alone. Recognition of these structural factors is essential when planning effective surgical correction.

Existing surgical options for LEME have several limitations. For example, the lazy-T procedure provides insufficient vertical lift. Transcaruncular medial canthal tendon plication is effective, but it involves deeper dissection and may pose a greater risk to the surrounding structures, including the canaliculi and medial rectus fascia [11,12]. The medial tarsal suspension technique, while effective for medial lower eyelid elevation, can be technically complex and carries the risk of recurrence [10]. The procedure most similar to the CMT technique reported in the present study is modified medial tarsorrhaphy, although it involves a more complex surgical technique [4].

The CMT technique promotes adhesion by removing the conjunctival epithelium, thereby generating the necessary tractional force for LEME correction. The connective tissue located beneath the caruncle is directly continuous with the lacrimal fascia and exerts a posterior vector of pull [1]. Accordingly, excising the conjunctiva medial to both the upper and lower puncta - including the caruncle - enables the creation of multidirectional traction by way of vertical and horizontal superomedial vectors that elevate and approximate the inferior punctum toward the superior punctum, and a posterior vector that repositions the everted punctum toward the ocular surface. This effect is clearly demonstrated in the representative postoperative photographs shown in Figures 2A-2C, 3A, 3B, 4A, 4B.

The CMT technique shortens the vertical punctal distance while minimizing the risk of tearing complications. No cases of postoperative epiphora were observed in the present study. Two cases of intraoperative canalicular epithelial injury were managed successfully with silicone tube intubation, and no cases of postoperative epiphora were observed.

Anatomical studies have shown that the vertical canaliculus is tightly integrated into the tarsal plate and surrounded by Riolan’s muscle, while the horizontal canaliculus is enveloped by Horner’s muscle, which plays a crucial role in the lacrimal pump mechanism during blinking [17,18]. Thus, preserving these structures during CMT likely contributes to the maintenance of tear drainage function.

This procedure intentionally creates a configuration resembling kissing puncta, in which the upper and lower puncta are in contact. Although kissing puncta, otherwise known as punctal apposition, has been reported as a cause of epiphora, as previously mentioned, no cases of postoperative epiphora were observed in the present study. Therefore, the kissing puncta configuration, although classically associated with epiphora, may not itself be causative. Recent insights suggest that the underlying etiologies of punctal malposition, such as medial eyelid laxity or orbicularis dysfunction, are more critical determinants of functional impairment than puncta approximation [19].

A previous report suggested that medial tarsorrhaphy has minimal impact on lacrimal pump function [2]. Moreover, modified medial tarsorrhaphy involves a similar surgical field and intraoperative manipulation to the CMT technique, and no postoperative worsening of epiphora has been reported. On the contrary, it has been suggested that by enhancing blink dynamics, the procedure may exert a favorable effect on lacrimal pump function.

CMT utilizes conjunctival adhesion. As with several other techniques, some degree of regression may occur; however, our study demonstrated that the CMT technique provided effective results from the first surgery and allowed for a second additional procedure to be performed using the same method. Even in revision cases, the same technique was safely used with good outcomes.

This study has several limitations. First, not all surgeries were performed by a single surgeon, which may have introduced inter-surgeon variability in technique and outcomes. Second, many cases involved concomitant procedures such as canthopexy or lateral tarsal strip, making it difficult to attribute all improvements in LEME correction solely to CMT. Nevertheless, because CMT was the only procedure involving direct medial manipulation, the improvement in punctal distance likely reflects a true effect of the CMT itself. Third, as a retrospective study with a relatively small sample size and variable follow-up durations, potential selection and observation biases cannot be excluded. Prospective studies with standardized surgical protocols and longer follow-up are required to confirm these findings.

## Conclusions

CMT is a minimally invasive, safe, and effective method for treating lower eyelid medial ectropion. It directly addresses the medial malposition that is often uncorrected by lateral tightening alone, achieving both functional and aesthetic improvement. Its simplicity and reproducibility make it a practical first-line surgical option for a wide range of etiologies. Further prospective studies with standardized protocols are warranted to confirm the long-term outcomes and to determine the isolated efficacy of CMT as a standalone procedure.
